# Measurement of acetylcholinesterase activity by electrochemical analysis method utilizing organocatalytic reactions

**DOI:** 10.1039/d5ra04585a

**Published:** 2025-09-09

**Authors:** Tetsuya Ono, Takumi Terasaki, Riho Domon, Otoha Miho, Kentaro Yoshida, Shigehiro Takahashi, Takenori Dairaku, Yoshitomo Kashiwagi, Katsuhiko Sato

**Affiliations:** a School of Pharmaceutical Sciences, Ohu University 31-1 Misumido, Tomita-machi Koriyama Fukushima 963-8041 Japan t-ono@pha.ohu-u.ac.jp; b Hydrogen Energy Research Institute, Fukushima University Fukushima City Fukushima 960-1296 Japan; c Faculty of Pharmaceutical Science, Tohoku Medical and Pharmaceutical University 4-4-1 Komatsushima, Aoba Sendai Miyagi 981-8558 Japan

## Abstract

An electrochemical method for measuring acetylcholinesterase (AChE) activity was developed using nortropine-*N*-oxyl (NNO), an organocatalyst. The increase in catalytic current as NNO oxidizes choline allowed real-time monitoring of the AChE hydrolysis reaction. Compared to conventional H_2_O_2_-based sensors, this method eliminates one reaction step, enabling more direct and real-time monitoring of enzymatic activity. Amperometric measurements enable AChE activity determination over a range of 50–2000 U L^−1^ and the limit of detection and limit of quantification in the low concentration range were calculated to be 14.1 U L^−1^ and 46.9 U L^−1^, respectively, with a correlation coefficient (*R*^2^) of 0.9989. These results demonstrate that serum cholinesterase measurement using this method can be utilized for various diagnoses, such as liver and heart diseases. Furthermore, given the relevance of AChE in neurotoxicity evaluation, diagnosis of neurological disorders such as Alzheimer's disease, and environmental toxicity monitoring, this method has diverse potential applications. Moreover, this approach can be extended to other enzymatic reactions, indicating its promise for various analytical and diagnostic applications.

## Abstract 2

An electrochemical method for measuring acetylcholinesterase (AChE) activity was developed using nortropine-*N*-oxyl (NNO), an organocatalyst.

## Graphical abstract

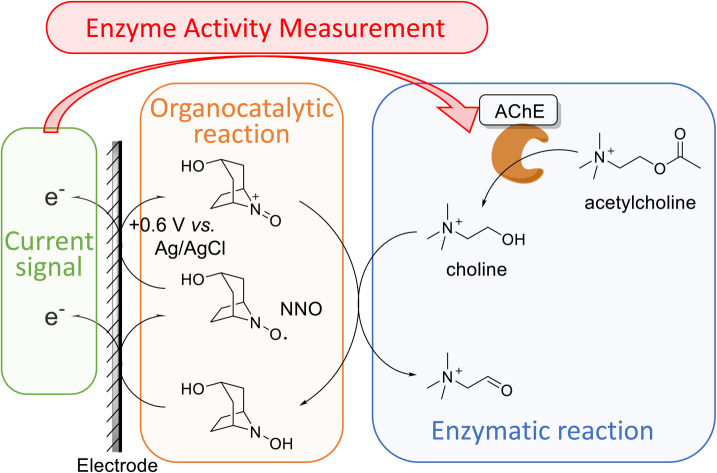

## Introduction

Nitroxyl radical compounds, represented by 2,2,6,6-tetramethylpiperidine 1-oxyl (TEMPO), have been used widely in various fields, including as oxidizing agents, battery materials, radical labeling agents, and fluorescent probes.^1–4^ In particular, TEMPO has been utilized in the field of electrochemistry as a catalyst for the electrochemical oxidation of organic compounds.^5,6^ Since the observed oxidation current is proportional to the concentration of the substrate, it has been demonstrated that TEMPO can be used as an analytical probe for electrochemical sensing of alcohols and other compounds containing hydroxy groups.^7,8^ However, the application of catalytic reactions using TEMPO is limited, nitroxyl radical compounds more reactive than TEMPO have been explored.^9–12^ We previously developed and reported the applicability of nortropine-*N*-oxyl (NNO), a highly active nitroxyl radical compound.^13–16^ NNO is capable of catalytically oxidizing molecules containing hydroxy or amino groups under physiological conditions. By monitoring the resulting catalytic current, compounds possessing these functional groups, such as glucose and pharmaceuticals, can be sensed electrochemically.^13–16^ Although this was difficult to achieve with TEMPO, it was made possible by two properties of NNO: the steric effect of reduced steric hindrance around the active site of the reaction provided by the bicyclo skeleton and the electronic effect of the intramolecular electron-withdrawing groups, which enable efficient alcohol oxidation.^17,18^

While NNO has the versatility to catalytically oxidize various compounds, its high reactivity makes it unsuitable for the selective detection of specific targets. To overcome this limitation, we combined an enzyme reaction with high substrate specificity and NNO-mediated catalytic oxidation to achieve selective quantification of target compounds.^19^ Specifically, we developed a highly sensitive and facile electrochemical detection method for triglycerides using only NNO and lipase as the enzyme. Conventional triglyceride sensors typically require three different enzymes; however, in this study, the reaction proceeds with lipase alone, enabling reduced cost and simplified operation.

In recent years, electrochemical sensing methods for enzyme activity measurement have been investigated.^20,21^ However, many of these methods rely on sensors that detect hydrogen peroxide (H_2_O_2_) generated as a product of enzymatic reactions. As a result, they are not suitable for reaction systems that do not produce H_2_O_2_ and often require multiple reaction steps to generate H_2_O_2_, which presents a significant limitation (see below). In contrast, electrochemical approaches using NNO allow the direct oxidation of substrates or products of enzymatic reactions to produce a current signal. This strategy effectively overcomes the limitations of conventional methods and provides a more direct and versatile solution for enzyme activity measurement.

In this study, we report the application of this method for the measurement of acetylcholinesterase (AChE, EC 3.1.1.7) activity. AChE is an essential enzyme that hydrolyzes the neurotransmitter acetylcholine, thereby terminating nerve signal transmission.^22,23^ Therefore, the measurement of AChE activity is crucial for understanding and assessing normal neurological function. For example, in neurodegenerative diseases such as Alzheimer's disease, therapeutic strategies aim to inhibit the degradation of acetylcholine to slow the progression of symptoms. Hence, the quantification of AChE activity is indispensable for evaluating the efficacy of AChE inhibitors and monitoring disease progression.^24,25^ In addition, AChE is irreversibly inhibited by organophosphorus and carbamate pesticides, as well as nerve agents such as sarin. As such, AChE activity measurement is used in toxicity assessment and diagnosis of poisoning.^26^ Furthermore, biosensors that detect AChE inhibition have been developed for identifying residual pesticides and neurotoxic substances in the environment, and are being applied to food and water safety assessment.^27,28^ AChE is also a target in pharmaceutical development, and the measured activity is a fundamental and important parameter for the screening of new AChE inhibitors for dementia treatments or insecticides, as well as for structure–activity relationship studies. Thus, AChE activity assays have been actively investigated due to their significant relevance across diverse fields such as neuroscience, drug development, toxicology, and environmental analysis.^29,30^

In conventional electrochemical methods for evaluating AChE activity, the change in current is detected by the oxidation or reduction of hydrogen peroxide generated through reactions (1) and (2) at the electrode surface.^31,32^

1 Acetylcholine → (AChE) → choline + acetic acid

2 Choline + O_2_ → (ChOx) → betaine + H_2_O_2_

**Fig. 1:**
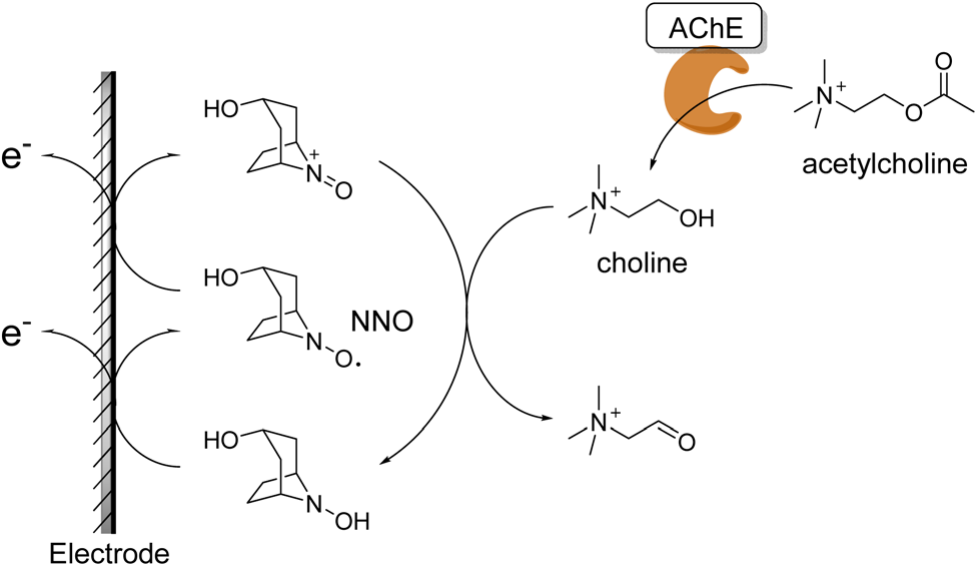
Principle of AChE activity measurement.

However, conventional methods face the drawback of requiring multiple enzymes that are expensive and unsuitable for long-term storage. The method proposed in this study addresses this issue. As shown in Fig. 1, acetylcholine is hydrolyzed by AChE into choline and acetic acid. The resulting choline is then non-enzymatically oxidized by NNO, and the oxidation current generated in this process increases proportionally to the choline concentration. This current response can be used as an indicator of AChE activity. In other words, this method enables a simpler and faster evaluation of AChE activity than conventional techniques by using only AChE as the enzyme and detecting the produced choline electrochemically *via* the stable organic molecular catalyst NNO.

## Experimental

### Materials

NNO was synthesized in a single step from nortropine according to a previously reported method.^13^ Nortropine was purchased from Tokyo Kasei, AChE (from Electrophorus electricus, Type VI-S, lyophilized powder, 200–1000 units per mg protein) from Sigma-Aldrich, acetylcholine chloride (≥98%) from FUJIFILM Wako Chemicals, and choline chloride (≥98%) from Nacalai Tesque. All other reagents used for synthesis were commercially available and used as received.

### Apparatus and methods

Amperometric measurements were conducted using an electrochemical analyzer (ALS model 440C, BAS, Tokyo, Japan). The measurements were conducted at 37 °C with stirring in a Water-Jacketed glass cell (20 mL, BAS, Tokyo, Japan) consisting of a glassy carbon working electrode (diameter: 3 mm), a platinum wire counter electrode, and an Ag/AgCl reference electrode (3 mol L^−1^ NaCl). Amperometric measurements were carried out in 10 mL phosphate buffer (100 mM, pH 7.4), and the applied potential was set to +0.6 V *vs.* Ag/AgCl, based on the standard redox potential of NNO (+0.53 V *vs.* Ag/AgCl), to ensure sufficient electrochemical oxidation of NNO.^15^ The difference in current (Δ*I*) before and after the addition of substrate or enzyme was used as the analytical parameter.

### Calculation of LOD and LOQ

The limit of detection (LOD) and limit of quantification (LOQ) were calculated using eqn (3) and (4), respectively.^33^3
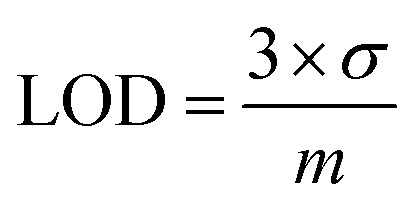
4
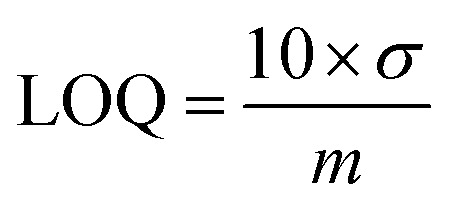


The symbol *σ* represents the standard deviation of noise at +0.6 V *vs.* Ag/AgCl in amperometry measurements, and *m* denotes the slope of the calibration curve.

## Results and discussion

**Fig. 2:**
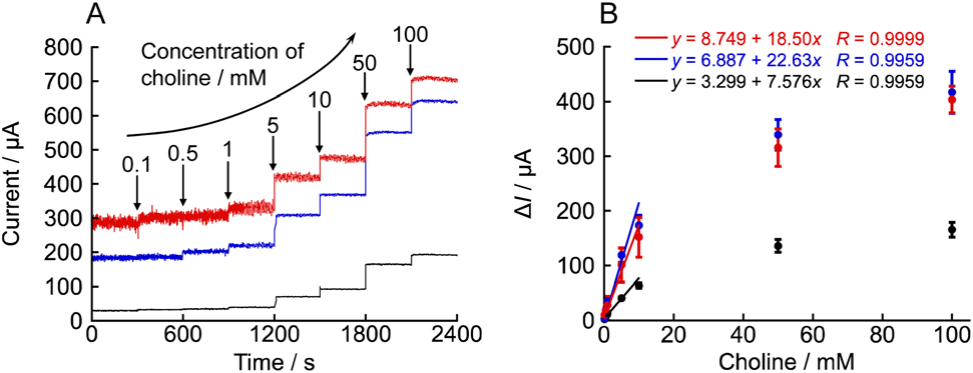
(A) Amperometric responses obtained using various concentrations of NNO (1 mM: black; 5 mM: blue; 10 mM: red) toward sequential additions of choline chloride at +0.6 V *vs.* Ag/AgCl in 100 mM phosphate buffer (pH 7.4). (B) Plots of current increase (Δ*I*) *versus* choline concentration (*n* = 3, mean ± SD).

The effect of NNO concentration on the choline detection was investigated using amperometry. The results of the comparison of anodic current values for 0.1–100 mM choline chloride solutions with 1, 5, and 10 mM NNO are shown in Fig. 2. As seen in Fig. 2A, the anodic current increased stepwise with increasing choline chloride concentration for all NNO concentrations. Upon addition of 100 mM choline chloride, the measured current values were 191.2, 640.8, and 703.9 μA for 1, 5, and 10 mM NNO, respectively, indicating that the current value increased with NNO concentration. Furthermore, as shown in Fig. 2B, when the change in current from that for the blank (Δ*I*) was used as the index of evaluation, the Δ*I* values for 100 mM choline chloride were 165.4, 416.6, and 403.7 μA for 1, 5, and 10 mM NNO, respectively, demonstrating that the response tended to saturate at NNO concentrations above 5 mM. (Note: since NNO itself is electrochemically oxidized at the electrode, the blank current increases with NNO concentration.) In contrast, using 1 mM NNO, even at low choline chloride concentrations (1–10 mM), the Δ*I* values ranged from 12.9 to 63.6 μA, showing a sufficiently wide response range with good reproducibility, with a standard deviation of less than 4.5 μA. From multiple measurements in the absence of choline, *σ* at NNO concentrations of 1, 5, and 10 mM were calculated to be 5.82 × 10^−7^, 3.90 × 10^−6^, and 5.51 × 10^−6^ A, respectively. Similarly, the slope *m* were determined to be 7.58 × 10^−3^, 2.26 × 10^−2^, and 1.85 × 10^−2^ A M^−1^, respectively (Fig. 2B). Therefore, at NNO concentrations of 1, 5, and 10 mM, the LOD 0.23, 0.52, and 0.89 mM, the LOQ 0.77, 1.73, and 2.98 mM were calculated, respectively.

**Fig. 3:**
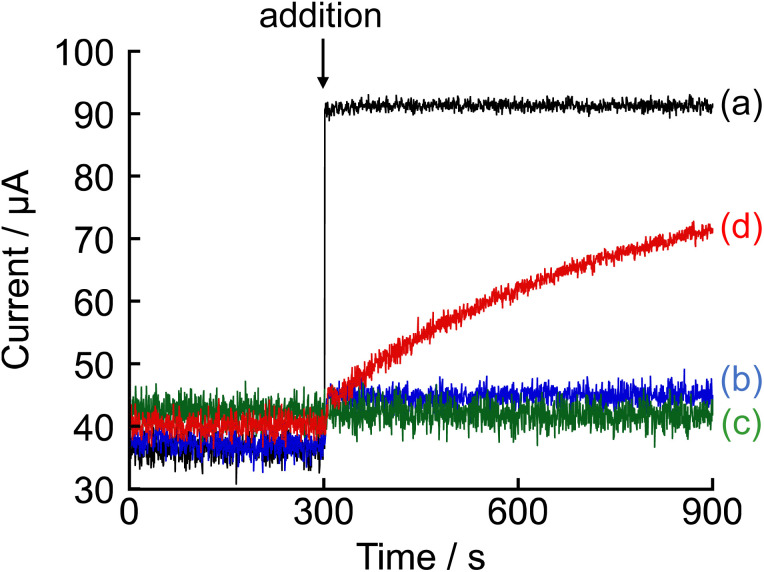
Amperometric responses obtained upon the addition of various analytes at 300 s into a 100 mM phosphate buffer (pH 7.4) containing 1 mM NNO. The applied potential was +0.6 V *vs.* Ag/AgCl. (a) Choline chloride (10 mM), black; (b) acetylcholine chloride (10 mM), blue; (c) AChE (500 U L^−1^), green; (d) acetylcholine chloride (10 mM) and AChE (500 U L^−1^), red.

The progression of the enzymatic reaction was investigated using amperometry. As shown in Fig. 3, measurements were conducted in 100 mM phosphate buffer (pH 7.4) containing 1 mM NNO, and the following were added individually at 300 s: (a) 10 mM choline chloride, (b) 10 mM acetylcholine chloride, (c) 500 U L^−1^ AChE, and (d) 10 mM acetylcholine chloride and 500 U L^−1^ AChE. Upon addition of choline chloride alone, a sharp increase in current was observed (Δ*I* = 55.0 μA). As shown in Fig. 2, this catalytic current is attributed to the oxidation of the hydroxy group of choline by NNO, indicating that this oxidation reaction proceeds rapidly under amperometric conditions. Therefore, this oxidation is not the rate-limiting step in the enzymatic activity measurement performed in this study. In contrast, when either acetylcholine chloride or AChE was added individually, no significant change in current was observed, confirming that no direct catalytic oxidation by NNO occurs under these conditions. Furthermore, when both acetylcholine chloride and AChE were added simultaneously, the current gradually increased over time, with a Δ*I* of 8.2 μA at 1 min after addition, reaching 31.2 μA at 10 min. These results suggest that choline generated by the hydrolysis of acetylcholine by AChE was rapidly oxidized by NNO. Taken together, these findings demonstrate that the increase in current observed under the condition where acetylcholine chloride and AChE were added together serves as a reliable indicator of the progress of the enzymatic reaction.

**Fig. 4:**
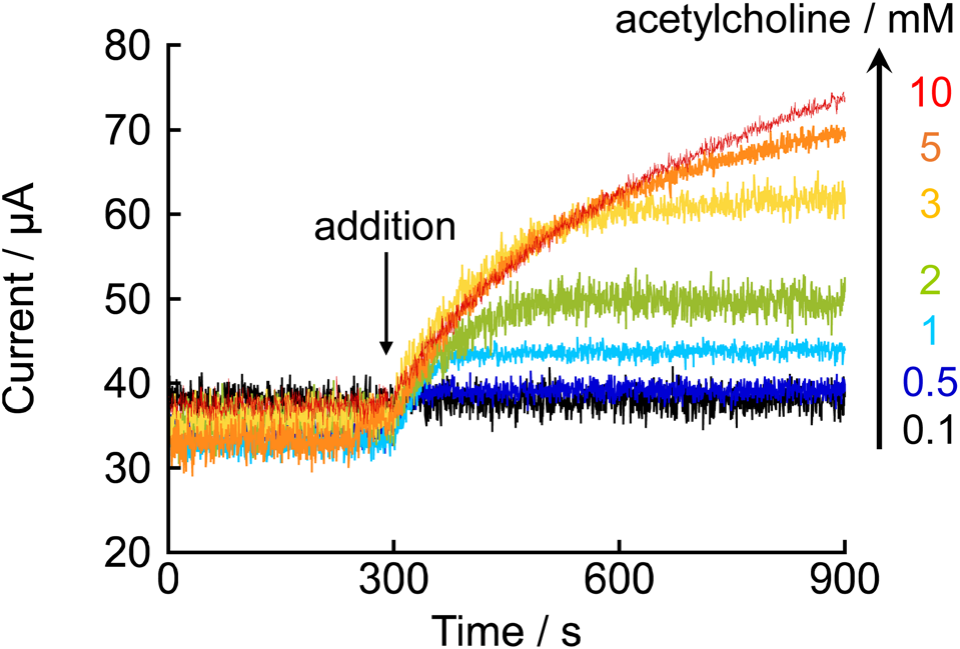
Amperometric responses obtained upon addition (at 300 s) of varying concentrations of acetylcholine chloride (0.1–10 mM) to a phosphate buffer (100 mM, pH 7.4) containing 1 mM NNO and 1000 U L^−1^ AChE. The applied potential was +0.6 V *vs.* Ag/AgCl.

Next, the optimal concentration of acetylcholine chloride for enzyme activity measurement was investigated. Amperometric measurements were performed using an electrolyte solution containing 1 mM NNO and 1000 U L^−1^ AChE, and the change in current was monitored following the addition of acetylcholine chloride (0.1–10 mM) at 300 s (Fig. 4). When acetylcholine chloride was added at concentrations of 3 mM or higher, a sharp increase in current was observed immediately after addition, and the initial response profile was similar across these concentrations. This suggests that the enzyme was already saturated with substrate at the early stage of the reaction, and that the reaction proceeded near the maximum enzymatic rate (*V*_max_). In contrast, under lower substrate concentrations (*e.g.*, 0.1–0.5 mM), the current reached a plateau around 39.3 μA, and the increase in current ceased within a short period. This is likely due to the rapid consumption of the substrate by the enzyme over time, preventing maintenance of a substrate concentration sufficient to reach *V*_max_. Based on these findings, to ensure an adequate substrate supply and stable measurement of enzyme activity, the concentration of acetylcholine chloride was set at 10 mM for subsequent experiments.

**Fig. 5:**
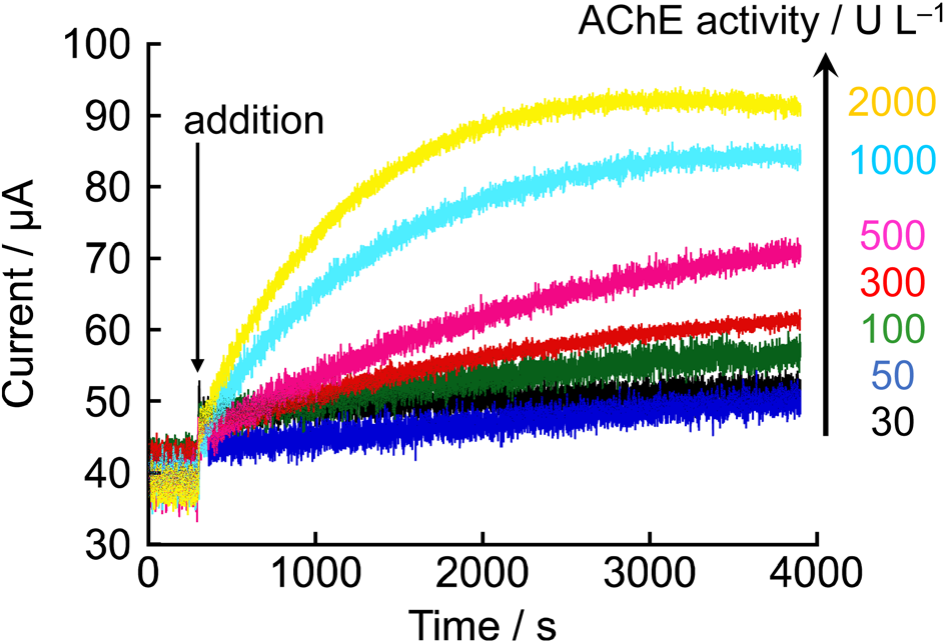
Amperometric responses obtained after the addition of acetylcholine chloride (10 mM) at 300 s to electrolyte solutions containing 1 mM NNO and varying concentrations of AChE (30, 50, 100, 300, 500, 1000, and 2000 U L^−1^). Measurements were performed at +0.6 V *vs.* Ag/AgCl in 100 mM phosphate buffer (pH 7.4).

Based on the results obtained thus far, it was confirmed that the progress of the enzymatic reaction can be evaluated from current values using NNO-based amperometry. Therefore, the measurement of AChE activity was performed using this system. Fig. 5 shows the amperometric responses obtained after adding 10 mM acetylcholine chloride at 300 s to electrolyte solutions containing 1 mM NNO and varying concentrations of AChE (30, 50, 100, 300, 500, 1000 and 2000 U L^−1^). Following the addition of acetylcholine, an increase in current was observed under all conditions; however, both the rate of increase and the final current value (after 1 hour) were dependent on AChE concentration. For example, the final current values at 1000 U L^−1^ and 500 U L^−1^ AChE were 84.3 μA and 70.9 μA, respectively, indicating that higher AChE concentrations resulted in a faster current rise and higher final values. In contrast, at lower AChE concentrations (30–100 U L^−1^), the current increase was relatively small and often obscured by background noise from the intrinsic oxidation of NNO, making it difficult to observe a clear current response proportional to enzyme activity. When using higher AChE concentrations (300 U L^−1^ and above), sufficiently distinguishable differences in current were observed, suggesting that AChE activity can be reliably measured within this range.

**Fig. 6:**
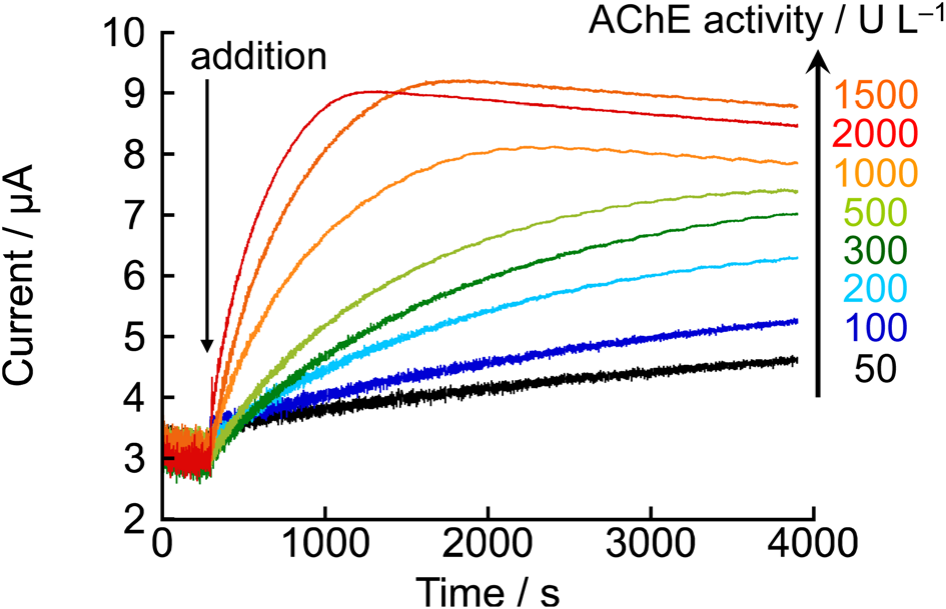
Amperometric responses obtained after the addition of acetylcholine chloride (10 mM) at 300 s to electrolyte solutions containing 0.1 mM NNO and various concentrations of AChE (50, 100, 200, 300, 500, 1000, 1500, and 2000 U L^−1^). Measurements were performed at +0.6 V *vs.* Ag/AgCl in 100 mM phosphate buffer (pH 7.4).

Although current changes dependent on enzyme activity were observed, significant variations were not obtained in the low activity range below 300 U L^−1^. Therefore, we thought that reducing the concentration of NNO and decreasing its own current value would allow for more distinct signal detection in the low activity range. To test this, the same experiment was conducted using 0.1 mM NNO (Fig. 6). As a result, clear differences in both the initial current rise and final current values were observed in the AChE activity range of 50–300 U L^−1^. At 60 min after acetylcholine addition, the current values for AChE concentrations of 50, 100, and 300 U L^−1^ were 4.6, 5.3, and 7.0 μA, respectively, showing a stepwise increase corresponding to the increase in enzyme activity. A clear, activity-dependent current response was successfully obtained. In particular, significant increases in current were observed even under low activity conditions such as 50 U L^−1^ and 100 U L^−1^, indicating that sufficient sensitivity and reproducibility were maintained even at 0.1 mM NNO. These findings further confirm that the oxidation of choline chloride by NNO is not the rate-limiting step, and that the observed current reflects the progress of the enzymatic reaction driven by AChE. This method was demonstrated to be capable of quantitatively tracking the enzymatic reaction even in the low-activity range below 300 U L^−1^, suggesting its potential application for monitoring in clinical diagnostics and pharmacological evaluations.

**Fig. 7:**
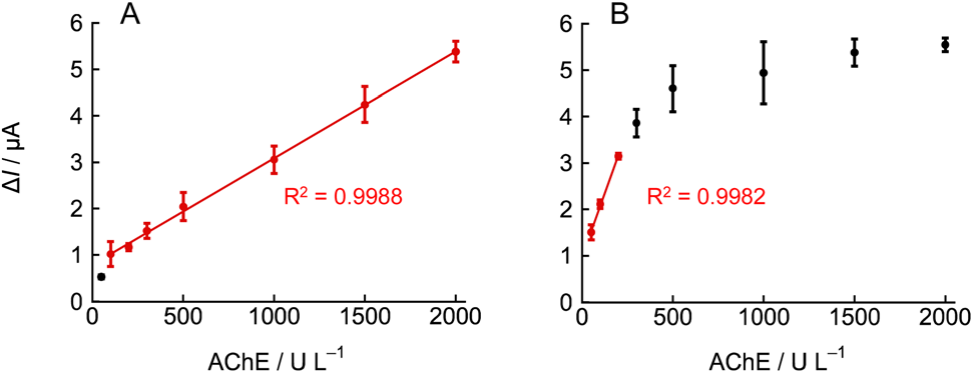
Calibration curves plotting the current increase (Δ*I*) *versus* AChE activity at (A) 10 min and (B) 60 min after the addition of 10 mM acetylcholine chloride, other conditions as in Fig. 6 (*n* = 3, mean ± SD).

By lowering the concentration of NNO, significant current changes were obtained even in the AChE activity range below 300 U L^−1^. Based on this, calibration curves for AChE activity were constructed using the current values at 10 and 60 min after acetylcholine addition from Fig. 6 (Fig. 7). When the current at 10 min after acetylcholine addition was used as the index, a good linear relationship (*R*^2^ = 0.9988) was observed within the AChE activity range of 100–2000 U L^−1^ (Fig. 7A). In contrast, when using the current at 60 min as the index, good linearity (*R*^2^ = 0.9982) was observed for AChE activity only below 200 U L^−1^ (Fig. 7B). At higher concentrations of AChE, the current tended to plateau, and the linear relationship between current and enzyme activity broke down. This is likely due to the rapid hydrolysis of acetylcholine for high enzyme activity, which caused the substrate concentration to drop below the level required to maintain *V*_max_ after a certain time. These results demonstrate that by adjusting the measurement time according to the target activity range, accurate quantification of AChE activity over a wide dynamic range is possible. Specifically, using the current at 10 min as the index resulted in excellent linearity over a broad range of enzyme activity, while using the 60-min current value allowed precise measurement in the low-activity region.

**Fig. 8:**
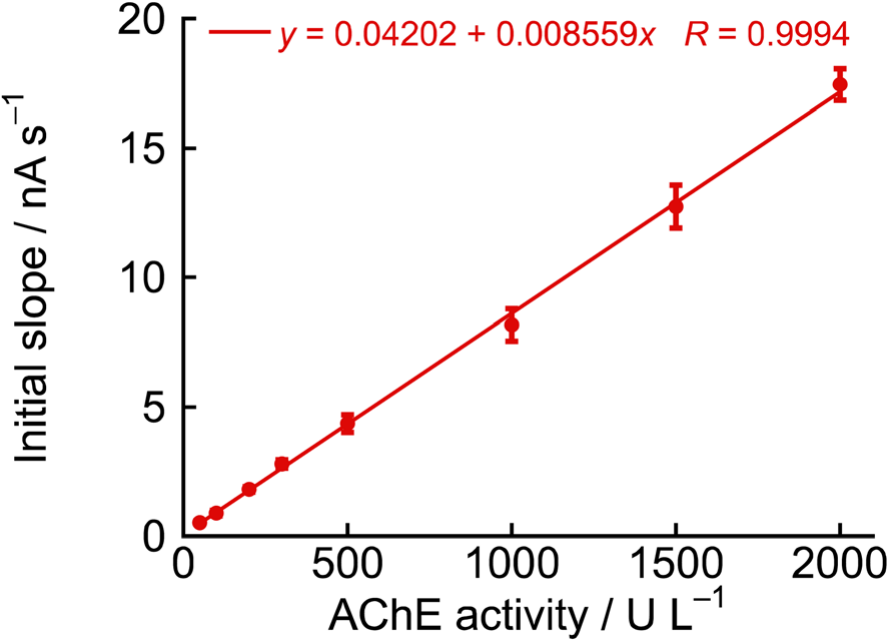
Calibration curves plotting the initial slope (for 1 min after acetylcholine chloride addition) of the current *versus* time curve in Fig. 6*versus* AChE activity, other conditions as in Fig. 6 (*n* = 3, mean ± SD).

Similarly, another approach to plotting the initial slope (for 1 min after acetylcholine chloride addition) of the current *versus* time curve (Fig. 6) was considered, and this method provided a superior calibration curve for AChE concentration in the range of 50–2000 U L^−1^ (Fig. 8). In this method, *σ* was calculated to be 7.23 × 10^−8^ A and the slope *m* was determined to be 8.56 × 10^−12^ A s^−1^ U^−1^ L. Therefore, the LOD and the LOQ were calculated to be 422.3 U L^−1^ and 1407.7 U L^−1^, respectively. This method showed low sensitivity due to high noise caused by stirring of the electrolyte. However, at levels below 300 U L^−1^, a linear increase in current values was observed even after 10 min following the addition of acetylcholine chloride. In addition, a similar linear increase was observed up to 30 min after the addition of acetylcholine at 50 U L^−1^. Therefore, when the slope was calculated based on the current values over a period of 30 min, the LOD and the LOQ were calculated to be 14.1 U L^−1^ and 46.9 U L^−1^, respectively. These results sufficiently cover the range of serum cholinesterase activity measurements for diagnosis of liver disease and assessment of mortality risk due to COVID-19 pneumonia.^34–36^ Therefore, this measurement method is considered a rapid and simple approach for evaluating AChE activity.

## Conclusions

In this study, the activity of acetylcholinesterase (AChE) was measured using an amperometric method based on the use of nortropine-*N*-oxyl (NNO) as an organocatalyst, and the utility of this approach was evaluated. First, it was confirmed that NNO promotes the electrochemical oxidation of choline chloride, and based on this property, it was demonstrated that the hydrolysis of acetylcholine by AChE could be monitored in real time. Measurements performed at varying AChE concentrations showed that the current value increased in proportion to enzyme activity, yielding a good linear relationship. These results suggest that this method is suitable for quantifying a broad range of AChE activities. While traditional methods for measuring AChE activity mainly rely on colorimetric or fluorometric techniques, the present method offers the advantages of rapid and simple evaluation through electrochemical measurement. Moreover, this approach is applicable to a variety of enzymatic reactions involving the formation or loss of hydroxy and amino groups, and is expected to be developed in the future.

## Author contributions

Conceptualization, T. O. and K. S.; methodology, T. O. and K. S.; software, T. O. and K. S.; validation, T. T., R. D., M. O., K. Y., S. T. and T. D.; formal analysis, T. O. and K. S.; investigation, T. T., R. D., M. O., K. Y., S. T. and T. D.; resources, T. O., Y. K. and K. S.; data curation, T. O. and K. S.; writing—original draft preparation, T. O. and K. S.; writing—review and editing, T. O. and K. S.; visualization, T. O. and K. S.; supervision, T. O. and K. S.; project administration, T. O. and K. S.; funding acquisition, T. O. All authors have read and agreed to the published version of the manuscript.

## Conflicts of interest

There are no conflicts of interest to declare.

## Data Availability

All the data that support the findings of this study are included in the manuscript.
